# Phylogenomic Analyses of the Hemagglutinin-Neuraminidase (*HN*) Gene in Human Parainfluenza Virus Type 4 Isolates in Japan

**DOI:** 10.3390/microorganisms13020384

**Published:** 2025-02-10

**Authors:** Kanako Otani, Ryusuke Kimura, Norika Nagasawa, Yuriko Hayashi, Suguru Ohmiya, Oshi Watanabe, Irona Khandaker, Hirokazu Kimura, Hidekazu Nishimura

**Affiliations:** 1 Center for Surveillance, Immunization, and Epidemiologic Research, National Institute of Infectious Diseases, Tokyo 162-8640, Japan; otanik@niid.go.jp; 2 Virus Research Center, Clinical Research Division, Sendai Medical Center, Sendai 983-8520, Japan; oomiya.suguru.qj@mail.hosp.go.jp (S.O.); oshiwat@gmail.com (O.W.); irk14@pitt.edu (I.K.); 3 Department of Bacteriology, Graduate School of Medicine, Gunma University, Maebashi-shi 371-8511, Japan; m2220015@gunma-u.ac.jp; 4 Advanced Medical Science Research Center, Gunma Paz University, Takasaki-shi 370-0006, Japan; 5 Department of Health Science, Graduate School of Health Sciences, Gunma Paz University, Takasaki-shi 370-0006, Japan; nagasawa@paz.ac.jp (N.N.); hayashi@paz.ac.jp (Y.H.); 6 Department of Surgery, Trauma and Transfusion Medicine Research Center (TTMRC), University of Pittsburgh, 200 Lothrop St., Pittsburgh, PA 15213, USA

**Keywords:** human parainfluenza virus type 4, *HN* gene/HN protein, phylogenomics

## Abstract

To better understand the phylogenomics of the hemagglutinin-neuraminidase (*HN*) gene and HN protein in human parainfluenza virus type 4 (HPIV4), we performed phylogenomic analyses using various bioinformatics methods. The main bioinformatics analyses included a time-scaled phylogeny, genetic distance assessments, and three-dimensional (3D) structure mapping of the HN protein with conformational epitope and selective pressure analyses. The time-scaled phylogenetic tree indicated that the most recent common ancestor of the *HN* gene emerged approximately 100 years ago. Additionally, the tree revealed two distinct clusters corresponding to HPIV4a and HPIV4b. The divergence times for the most recent common ancestors of the *HN* gene in HPIV4a and HPIV4b strains were estimated to be around 1993 and 1986, respectively. The evolutionary rates of the gene varied significantly between clusters, ranging from approximately 1.2 × 10^−3^ to 8.7 × 10^−4^ substitutions per site per year. Genetic distances within each cluster were relatively short (less than 0.04). Phylodynamic analyses demonstrated an increase in the genome population size around the year 2000. Structural analyses revealed that the active sites of the HN protein were located at the protein’s head. Furthermore, the most conformational epitopes were located in adjacent active sites of the protein. These results suggested that reinfection may be unlikely to occur in the case of most HPIV4. Together, the *HN* gene and protein of HPIV4 strains isolated in Japan have undergone unique evolutionary changes. In addition, antibodies targeting the conformational epitopes of the HPIV4 HN protein may contribute to protection against the virus.

## 1. Introduction

Human parainfluenza virus type 4 (HPIV4), also known as human rubulavirus type 4, belongs to the family Paramyxoviridae and the genus *Orthorubulavirus* [1]. It can cause various respiratory diseases, including the common cold, croup, bronchitis, and pneumonia [2]. Previous studies have classified HPIV4 into two subtypes, HPIV4a and HPIV4b, based on serological and phylogenetic analyses [3]. Epidemiological data suggest that the most prevalent types are HPIV3 (also known as human respirovirus type 3, HRV3) and HPIV1 (also known as human respirovirus type 1, HRV1), which belong to a different genus, *Respirovirus* [4,5]. However, the epidemiological profiles of HPIV2 and HPIV4 remain largely unknown because of the small case numbers reported in clinics for these two types [6,7]. Additionally, other epidemiological studies indicate that HPIV4, similarly to other respiratory viruses, such as human respiratory syncytial virus (HRSV) and influenza viruses, can cause reinfections, although this has not been fully addressed in previous research [8,9]. Clinically, human parainfluenza viruses (HPIVs) are commonly associated with acute upper respiratory tract diseases. However, in cases of lower respiratory tract illness or croup, a hospitalization rate of up to 17% has been reported among infected children under 5 years of age. Croup can occur with all HPIV types but is particularly prevalent in children infected with HPIV1 and HPIV2. In contrast, bronchiolitis is frequently observed in infants with HPIV3 infection. While HPIV4 typically causes only mild illness, it has also been reported to cause lower respiratory tract infections in some cases [10,11,12].

HPIV4 virions contain two major surface antigens: hemagglutinin-neuraminidase (HN protein) and fusion (F) protein [13]. The HN protein has dual functions, exhibiting both hemagglutinin and neuraminidase activities [14]. These activities play essential roles not only in the infection of host cells, but also in the budding process from infected cells [9,14]. Moreover, some monoclonal antibodies targeting the HN protein can neutralize the virus, thereby inhibiting infection of host cells [15,16]. These functions may be associated with the structure and evolution of the HN protein [17]. However, phylogeny, antigenicity, and the detailed molecular structure/function of the HN protein remain poorly understood.

Phylogenomic analyses using advanced bioinformatics technologies enable the study of evolutionary relationships among biological entities, including various viruses [18,19]. Phylogenomics involves analyzing viral RNA or protein sequence data to uncover the evolutionary history and connections between different organisms, providing insights into how they have evolved and diversified [20]. In this study, we performed phylogenomic analyses of the *HN* gene/HN protein in HPIV4 isolates from Japan to elucidate their evolutionary relationships.

## 2. Materials and Methods

### 2.1. Sample Collection, Virus Isolation, and Ethics Status

Clinical samples, including nasal swabs, nasopharyngeal swabs, and pharyngeal swabs, were collected when the patients visited the pediatric department at National Hospital Organization Sendai Medical Center or Nagai Pediatric Clinic in Sendai City for respiratory symptoms from July 2002 to December 2015. Additionally, samples were sent to the Sendai Medical Center from medical institutions in Japan for virus identification during the same period. The samples were stored in virus transport medium (Eagle MEM, 0.5% gelatin, 500 µg/mL streptomycin, 100 units/mL penicillin G) at 4 °C and transported to the virus research center. Virus isolation was performed using the microplate method [21]. Briefly, cell culture microplates containing human embryonic fibroblast (HEF), HEp-2, Vero, MDCK, and LLC-MK2 cells were prepared. The supernatant of the virus transport medium was inoculated into the microplates and incubated for several days. When specific cytopathic effects (CPEs) were observed in LLC-MK2 cells, the supernatant was harvested and stored at −20 °C. All samples were subjected to virus isolation for diagnostic purposes. Oral consent was obtained from the patients or their guardian(s) prior to the examination, and the test samples were collected. The implementation of this study, including the fact that written consent was not obtained, was reviewed and approved by the ethics committee. In addition, an opt-out procedure was also implemented. As a result, the study protocol was approved by the Ethics Committee on Human Research of National Hospital Organization Sendai Medical Center (approval Nos. 24-80, 24-81) on 15 October 2024, and the protocols were carried out in accordance with the approved guidelines.

### 2.2. RNA Extraction, Reverse-Transcription PCR, and Sequencing

The RNA of isolated viruses was extracted using the Viral RNA Mini Kit (QIAGEN, Hilden, Germany). Complementary DNA (cDNA) was synthesized with random primers. Polymerase chain reaction (PCR) targeting the *P* gene of PIV4 was performed to confirm the isolates as PIV4 [22]. The extracted RNA and cDNA were stored at −20 °C. The isolates confirmed for PIV4 were further analyzed using RT-PCR for the HA region. The PCR was carried out using the PrimeScript One Step RT-PCR Kit (TaKaRa, Kusatsu, Japan), in a reaction mixture containing 3 μL of RNA, 0.4 μM of forward and reverse primers, 12.5 μL of 2 × RT-PCR buffer, and 1.0 μL of enzyme mixture, with a final volume of 25 μL. The primer pairs and the product sizes are described elsewhere [23]. The reaction conditions included an initial incubation for 30 min at 50 °C, followed by 2 min at 94 °C. The cycling program consisted of 30 cycles of 30 s at 94 °C, 30 s at 45 °C, and 1 min and 30 s at 72 °C, with a final extension step at 72 °C for 7 min, using a thermal cycler (BIORAD, Hercules, CA, USA). The PCR products were analyzed by gel electrophoresis on 2% agarose with a 100 base-pair (bp) ladder. After purification using the Illustra ExoStar Kit (GE Healthcare, Chicago, IL, USA), the amplicon concentrations were quantified using a NanoDrop spectrophotometer (Thermo Fisher, Waltham, MA, USA). Sequencing was performed by Eurofins Operon (Japan) using the same primers used for PCR (Supplementary Table S1). Subtyping was conducted using a BLAST search, and a total of 140 strains were classified in this process.

### 2.3. Strains in the Present Study

To analyze the molecular evolution of the HPIV4 *HN* gene, we downloaded reference strains of HPIV4a and HPIV4b subtypes from GenBank [https://www.ncbi.nlm.nih.gov/genbank/; last accessed on 23 July 2024] (4a: MH892407 and KY460515, 4b: EU627591 and MN306032). A total of 129 strains, including these reference strains, were aligned using MAFFT software version 7 [24]. Irregular strains were excluded, resulting in a final dataset of 113 strains, including the reference strains. Details of the strains used in this study are shown in Supplementary Table S2.

### 2.4. Time-Scaled Phylogenetic Analyses

To estimate the phylogenetic relationships of the present strains, we constructed a phylogenetic tree using the Bayesian Markov Chain Monte Carlo (MCMC) method in the BEAST 2 package (v.2.6.7) [25]. First, we used jModelTest to determine the appropriate substitution model, selecting the TIM-Γ model. Next, we used the path-sampling/stepping-stone sampling marginal likelihood estimation method to select the best fit among four clock models (strict clock, relaxed clock exponential, relaxed clock log-normal, and random local clock) and two prior tree models (coalescent constant population and coalescent exponential population). These analyses were performed independently for all strains, and the relaxed clock log-normal along with the coalescent exponential population model were selected for the molecular evolutionary analysis. The effective sample size (ESS) was estimated using Tracer (v.1.7.2), and an ESS greater than 200 was confirmed for all parameters [26]. After applying a 10% burn-in, the phylogenetic tree was generated using TreeAnnotator (v2.6.7) and visualized with FigTree (v1.4.0). Evolutionary rates were estimated using the models selected for each dataset, as described previously. Statistics were analyzed using the Bonferroni method and Kruskal–Wallis test with EZR [27]. A *p*-value of less than 0.001 was considered statistically significant.

### 2.5. Phylogenetic Distance Analyses

To evaluate the phylogenetic distance among the present strains, we constructed the Maximum Likelihood tree using MEGA software version 7 and estimated it using the Patristic program [28]. In addition, to estimate the best substitution models, we used jModelTest2. Statistics were analyzed using the Bonferroni method and Kruskal–Wallis test with EZR [27]. A *p*-value of less than 0.001 was considered statistically significant.

### 2.6. Phylodynamic Analyses

To calculate the phylodynamics of the strains in this study, the effective population sizes (EPS) of the *HN* gene were estimated using Bayesian skyline plot (BSP) analysis with the BEAST package. The best models were estimated as previously described. The estimation for HPIV4b/II could not be made due to the small number (six strains). The BSP and the 95% highest probability density (HPD) intervals were visualized using Tracer.

### 2.7. Selective Pressure Analyses

To estimate positive or negative selection in the HN protein of the strains used in this study, we calculated the non-synonymous (*dN*) and synonymous (*dS*) substitution values at each codon position using the Datamonkey package 2.0 (last accessed on 20 October 2024). We applied the fixed effects likelihood (FEL), internal fixed effects likelihood (IFEL), single likelihood ancestor (SLAC), and fast unconstrained Bayesian approximation (FUBAR) methods. Positive selection (*dN*/*dS* > 1) and negative selection (*dN*/*dS* < 1) were identified based on *p*-values < 0.05 for SLAC, FEL, and IFEL, and posterior probabilities > 0.9 for FUBAR.

### 2.8. Construction of the 3D Structure of HN Proteins

In order to assess the relationship of amino acid substitutions between the strains in this study, we constructed three-dimensional structural models of the HN protein for each lineage using homology modeling. The 4JF7 (PDB ID) searched by protein BLAST was used as the template. The PDB file and the FASTA file were downloaded from the Protein Data Bank (PDB) (last accessed on 20 October 2024). The recent strain for each lineage was extracted; however, in cases where the dates overlapped, the selection was performed randomly. These amino acid sequences were aligned using MAFFTash, and the models were constructed with MODELER v.10.4 [29]. The optimal models for each lineage were selected based on Ramachandran plot analysis using WinCoot v.0.9.4.1 [30]. The best models were subjected to energy minimization using GROMOS96 within the Swiss–PDB Viewer software v4.1.0 [31].

### 2.9. Prediction of the Conformational Epitope

To evaluate the conformational B-cell epitopes of the constructed HN protein models, we applied SEPPA 3.0 [URL; http://www.badd-cao.net/seppa3/; last accessed on 24 October 2024] method with a cutoff value of 0.064 [32]. Regions with contiguous amino acid sequences, predicted by SEPPA 3.0 to contain more than three residues, were identified as conformational epitopes. These epitopes were subsequently mapped onto the constructed HN protein models.

## 3. Results

### 3.1. Time-Scaled Phylogeny and Evolutionary Rates

**Figure 1:**
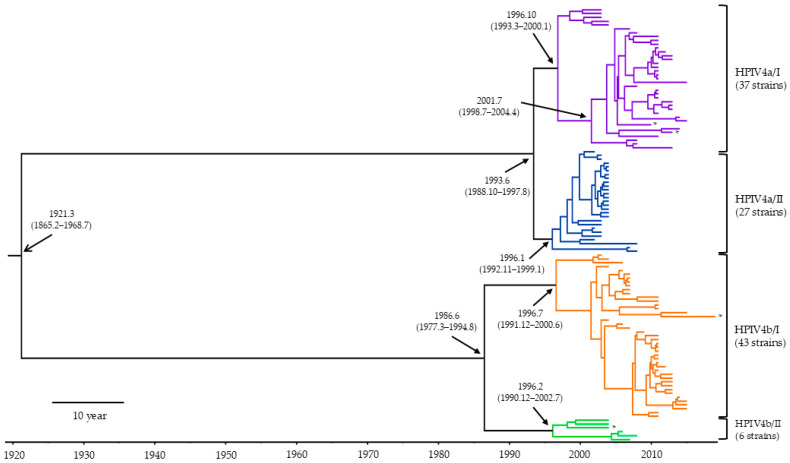
The time-scaled phylogenetic tree of the *HN* gene in HPIV4 was constructed using the Bayesian MCMC method. The divergence times with 95% highest probability densities (95% HPDs) are shown in the phylogenetic tree. Four asterisks (*) indicate the reference strains used in this study.

**Figure 2:**
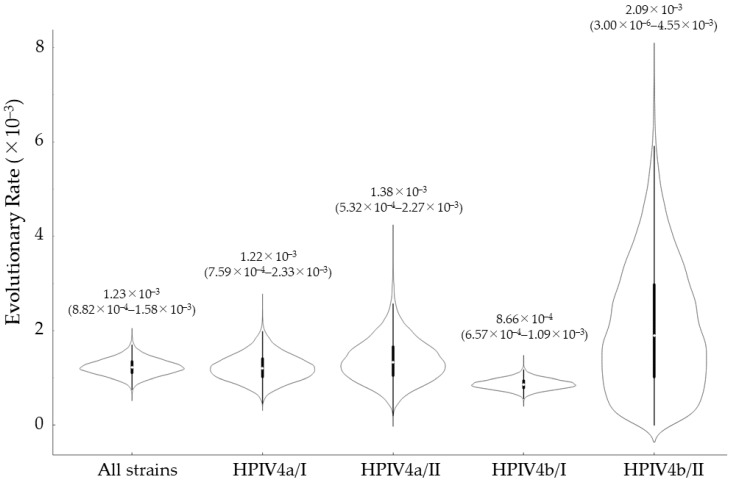
Evolutionary rates of the *HN* gene in HPIV4 were estimated using the Bayesian MCMC method. The rates with 95% HPDs are shown in the figure. The width of the violin plot represents the kernel density, indicating the shape of the data distribution. The central thick black bar and the thin black line represent the interquartile range and the full data range, respectively. The white dot indicates the median.

To estimate the phylogenetic evolution of the HPIV4 *HN* gene, we constructed a time-scaled phylogenetic tree and calculated the evolutionary rates using the MCMC method (Figure 1 and Figure 2). Initially, it was estimated that the common ancestor of the present strains emerged around March 1921 (mean; 95% HPDs, 1865.2–1968.7). It was further estimated that this common ancestor diverged into two subtypes (HPIV4a and HPIV4b). The common ancestor of HPIV4a and HPIV4b diverged around June 1993 (mean; 95% HPDs, 1988.10–1997.8) and June 1986 (mean; 95% HPDs, 1865.2–1968.7), respectively, and subsequently formed two lineages (HPIV4a/I, HPIV4a/II, and HPIV4b/I, HPIV4b/II). The evolutionary rate of the present strains was estimated to be approximately 1.23 × 10^−3^ substitutions per site per year (mean; 95% HPDs, 8.82 × 10^−4^–1.58 × 10^−3^). The evolutionary rates for the four lineages (HPIV4a/I, HPIV4a/II, HPIV4b/I, and HPIV4b/II) were estimated to be 1.22 × 10^−3^, 1.38 × 10^−3^, 8.66 × 10^−4^, and 2.09 × 10^−3^ substitutions per site per year, respectively. The evolutionary rates of each lineage showed statistically significant differences compared to the other lineages (*p* < 0.001). Detailed 95% HPDs for each lineage are provided in Figure 2. These results suggested that the lineages in the present strains evolved at different evolutionary rates, independently.

### 3.2. Phylogenetic Distance of the HN Gene in HPIV4

**Figure 3:**
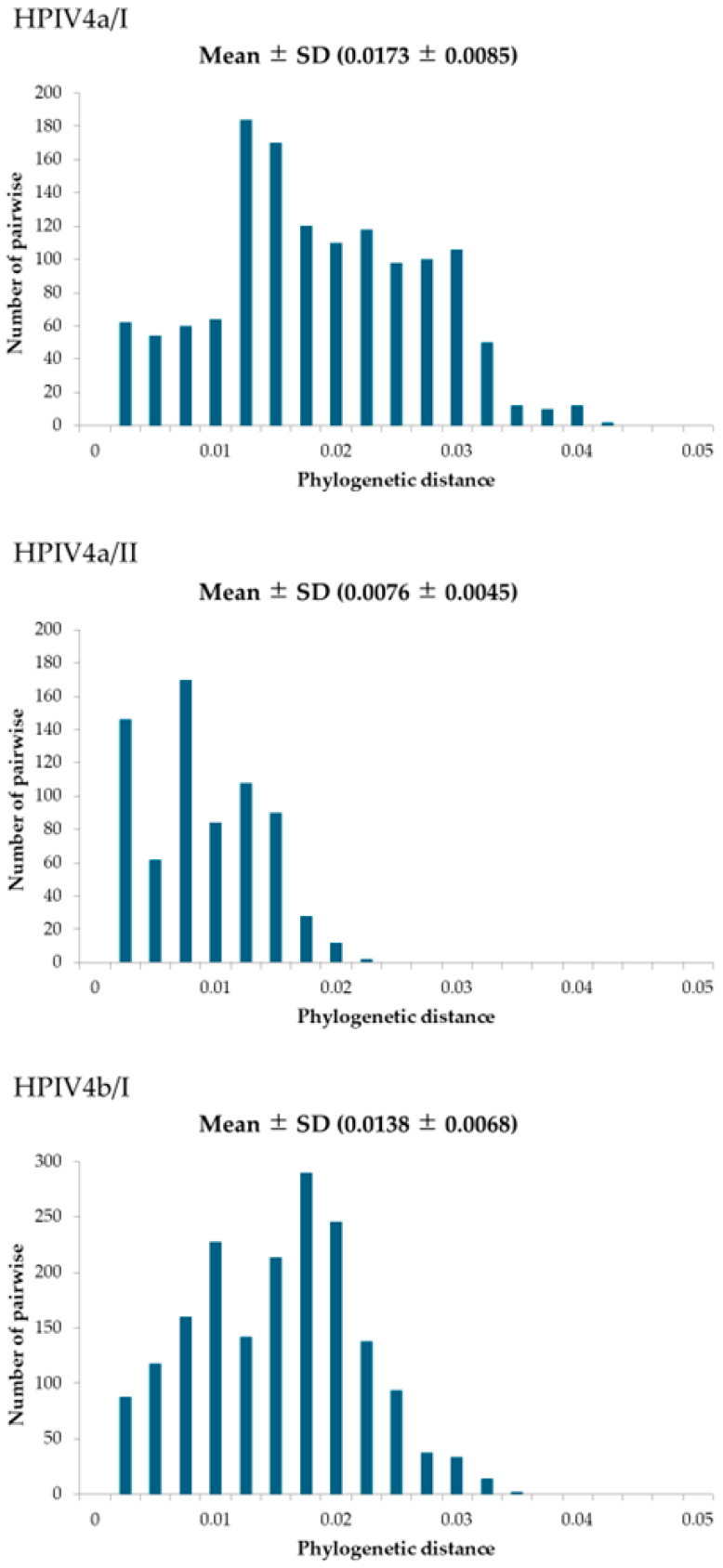

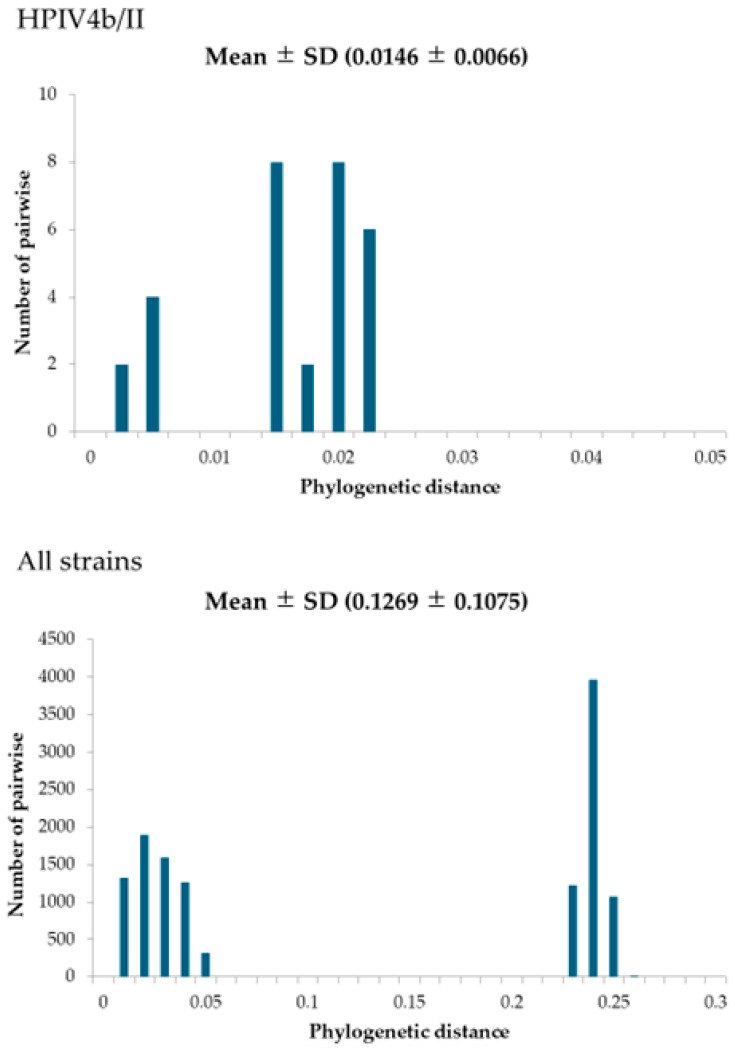
The phylogenetic distance of the *HN* gene in HPIV4 is represented as a bar graph. The vertical axis indicates the number of pairwise comparisons, and the horizontal axis indicates the phylogenetic distance. The mean and standard deviation of the phylogenetic distances are indicated in the figure.

In this study, to evaluate the genetic distance of the HPIV4 *HN* gene, we calculated phylogenetic distance using MEGA7 software (Figure 3). Firstly, the phylogenetic distances for the four lineages (HPIV4a/I, HPIV4a/II, HPIV4b/I, and HPIV4b/II) were as follows: 0.0173 ± 0.0085 (mean ± SD), 0.0076 ± 0.0045, 0.0138 ± 0.0068, and 0.0146 ± 0.0066, respectively. The phylogenetic distance of each lineage showed statistically significant differences compared to the other lineages (*p* < 0.001). Furthermore, when all strains were compared, a bimodal graph was obtained. This result suggests evolutionary differences, indicating that HPIV4a and HPIV4b have each experienced independent evolution.

### 3.3. Phylodynamics of the HN Gene in HPIV4

**Figure 4:**
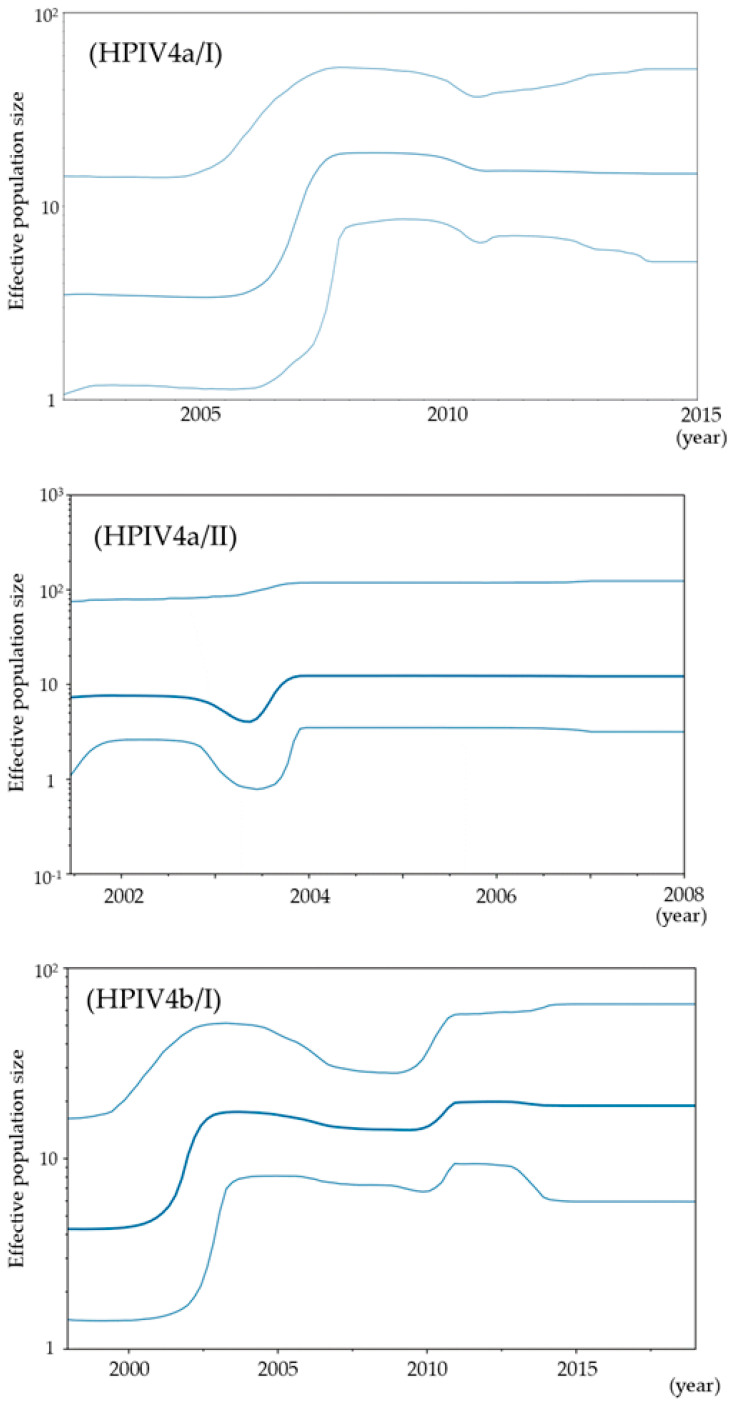

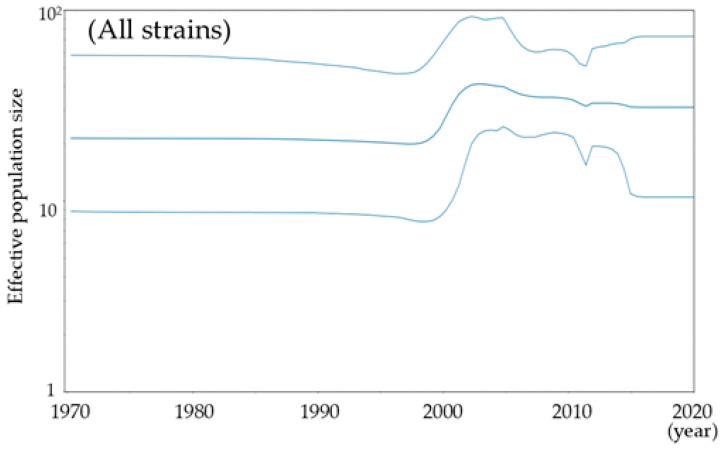
Phylodynamics of the present *HN* gene were calculated using Bayesian skyline plot analysis. The *x*-axis represents time (years), and the *y*-axis shows the effective population size. The thick central line indicates the median effective population size, while the thin lines above and below represent the 95% HPDs.

To assess the phylodynamics of the present strains, we estimated the effective population size using the BSP method (Figure 4). In HPIV4a/I, the effective population size increased around 2006 and has remained constant since 2008. In HPIV4a/II, both a drop and an increase were observed in 2003, but no significant change occurred in the effective population size (EPS), which has remained nearly constant. In HPIV4b/I, an increase in effective population size was observed around 2002 and has remained nearly constant since 2003, despite slow fluctuations. For all strains, an increase in effective population size was observed around 2000, after which it has remained nearly constant.

### 3.4. Relationships Between Selective Pressure and Active Sites of the HN Protein in HPIV4

**Figure 5:**
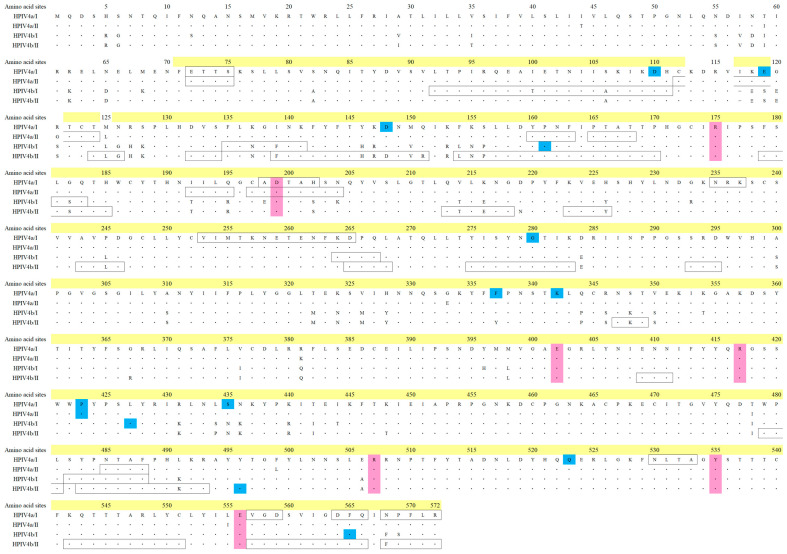
The HN protein amino acid sequences of HPIV4 were investigated in this study. The amino acid sequences were aligned based on PDB data for recent strains from each lineage, and the amino acid positions were indicated. Black line boxes in the sequence represent the conformational epitopes. Negative selection sites and the active site are highlighted in blue and pink, respectively. Amino acid positions highlighted in yellow indicate the portion used to construct the three-dimensional structure.

To determine the selective pressure on the host, positive and negative selection sites in the HN protein of total HPIV4 were predicted using the Datamonkey package. Several negative selection sites were predicted, but no positive selection sites were identified (Figure 5). No selective pressure was identified near the active site. More details on the active site relationships of influenza and other Paramyxoviridae are provided in Supplementary Table S3. These results suggested that the HN protein of HPIV4 may be less susceptible to selection pressure.

### 3.5. Relationships Between HN Protein Active Sites and Conformational Epitopes

**Figure 6:**
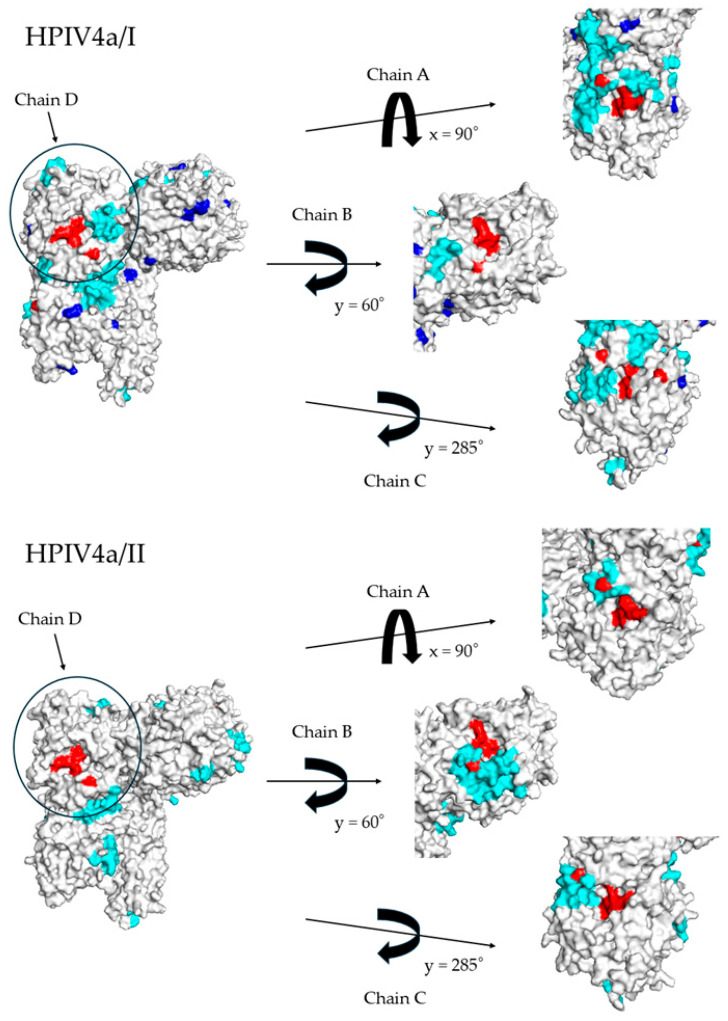

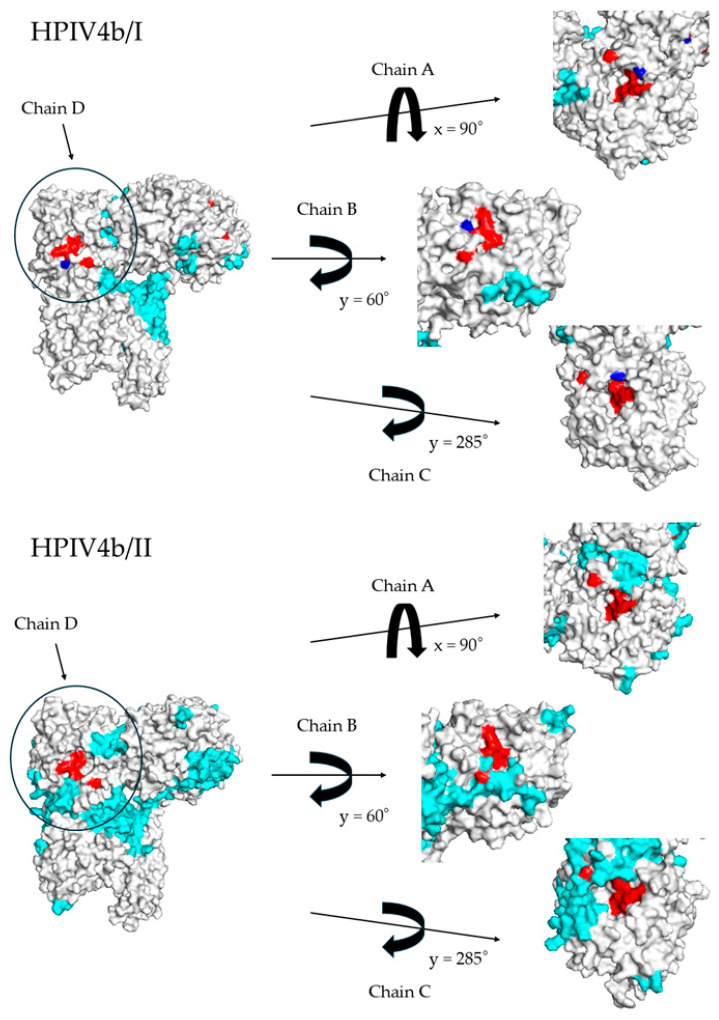
Three-dimensional (3D) structure and mapping of the HN protein in HPIV4. The negative selection sites and active sites of the HN protein are shown in blue and red, respectively. The conformational epitopes are colored in cyan. In cases of overlap, the color assignment follows this priority: active sites, conformational epitopes, and negative selection sites.

To understand the relationships between active sites and conformational epitopes on the HN protein, we constructed a 3D structure of the protein and mapped them. As shown in Figure 6, this protein formed a tetramer and exhibited a unique asymmetric structure. By rotating the viral particle and setting the D chain as a reference to bring the HN protein to the center front, it was observed that the active site of the HN protein on the A chain was positioned in a location visible from almost directly above the viral particle. However, the active sites of the HN proteins on the B and C chains were found to be similar to that of the A chain. Structurally, for HN proteins other than those in HPIV4b, conformational epitopes are positioned near or overlap with the HN active sites. These findings demonstrate that the HN protein activity of HPIV4 has a unique three-dimensional orientation. Additionally, antibodies induced by the conformational epitopes may inhibit HN activity, thereby suppressing intercellular infection, except in the case of HPIV4b. Moreover, the HN protein is less susceptible to positive selective pressure from the host.

## 4. Discussion

Most studies regarding the *HN* gene in HPIV focus on HPIV1 or HPIV3, while there are few studies on HPIV2 and HPIV4 [33,34,35,36,37,38,39]. Therefore, to better understand the molecular genetics of human parainfluenza virus type 4 (HPIV4), we performed phylogenomic analyses of the major antigen from 125 virus strains isolated from patients with respiratory illnesses in Japan. A time-scaled phylogenetic tree estimated that the most recent common ancestor of the *HN* gene emerged approximately 100 years ago (Figure 1). The tree also revealed two distinct clusters for both HPIV4a (clusters 4a/I and 4a/II) and HPIV4b (clusters 4b/I and 4b/II). The divergence times for the most recent common ancestors of the gene in the HPIV4a and HPIV4b strains were estimated to be around 1993 and 1986, respectively (Figure 1). The evolutionary rates of the gene in each cluster were significantly different, ranging from approximately 1.2 × 10^−3^ to 8.7 × 10^−4^ substitutions per site per year (Figure 2). Phylogenetic distance analyses indicated that the genetic distances of the *HN* gene within each cluster were relatively short, showing less than 0.04 (Figure 3). Next, phylodynamic analyses showed an increase in the genome population size of the gene around the year 2000 (Figure 4). Moreover, while these epitopes were located near the active sites in the HPIV4b protein, they were not found in similar proximity in the HPIV4a protein. Thus, antibodies induced by the conformational epitopes of the HPIV4b HN protein may contribute to protection against this virus. These results indicated that the *HN* gene in HPIV4 isolated in Japan has uniquely evolved over 100 years. To the best of our knowledge, these findings may be the first regarding the *HN* gene in HPIV4 isolated in Japan.

To estimate the time-scaled phylogeny of the *HN* gene of HPIV4 isolates in Japan, we constructed a phylogenetic tree using the Bayesian Markov Chain Monte Carlo method [25,40]. The results showed that the common ancestor of the *HN* gene in these isolates dated back over 100 years ago. Subsequently, its progeny diverged into two major lineages, resulting in HPIV4a and HPIV4b. Furthermore, HPIV4a and HPIV4b diverged after the 1980s or 1990s, forming two distinct clusters each. On the phylogenetic tree, both HPIV4a/I and 4b/I were estimated to be the prevalent types (Figure 1). A previous study showed that the common ancestor of the *F* gene (another major antigen-coding gene) of HPIV4, detected in various countries, dated back to approximately 200 years ago [13]. The phylogenetic topologies between the present and previous phylogenetic trees were significantly different, although the used strains were distinct [13]. This indicates that the *HN* and *F* genes in HPIV4 evolved independently, despite the use of distinct strains. Additionally, we estimated the evolutionary rate of the *HN* gene to be approximately 1.2 × 10^−3^ to 8.7 × 10^−4^ substitutions per site per year (Figure 2). Among them, the strain belonging to HPIV4a/II exhibited the fastest rate of evolution, although the reason is not known. A previous study assumed the evolutionary rate of the *F* gene to be 4.41 × 10^−4^ substitutions per site per year [13]. Therefore, the evolutionary rates of the *HN* and *F* genes are relatively similar. These findings suggest that the *HN* gene of HPIV4 isolates in Japan has evolved uniquely and acquired diversity over the past 100 years.

Next, we calculated the phylogenetic distance to evaluate the diversity among the strains (Figure 3). Long phylogenetic distances (over 0.2) were observed between the *HN* genes of HPIV4a and HPIV4b. However, the distances among lineages were relatively short (less than 0.04). Previous reports on the *F* gene of the virus also showed similar data [41]. Therefore, although genetic divergences are evident, the genetic distances for both major antigen-coding genes (*F* and *HN* genes) may remain relatively short [17,41].

We also examined the phylodynamics of the *HN* gene in HPIV4 (Figure 4). The results showed an increase in the genome population size of the *HN* gene in HPIV4a and 4b/I after 2000. A previous study on the *F* gene of HPIV4 based on globally collected data demonstrated differing phylodynamics. Specifically, the genome population sizes of HPIV4 and HPIV4a remained constant, while a notable increase (from approximately 20 to around 50) in the genome population size of HPIV4b was observed after 2010 [13]. The difference in the results of the phylodynamic analysis might be attributable to the difference in the epidemic dynamics of HPIV4, which may have varied between Japan and other regions of the world, or to a mere increase in viral detection bias with more frequent trials that started around the time in Japan. The present study became possible by utilizing regional strains that have become available since 2000 with the development of the isolation system, and this may represent a limitation of the study.

Next, we compared the catalytic amino acid residues in the HN proteins among HPIV4, influenza virus, HPIV3, and Newcastle disease (ND) virus (Table S3). Interestingly, a detailed amino acid sequence alignment analysis revealed that the amino acid residues of influenza virus, HPIV3, and Newcastle disease (ND) virus involved in neuraminidase activity are identical (Arg (R), Tyr (Y), Glu (E), and Asp (D)), although structural differences exist in the HN proteins among these viruses. Based on these results, it is estimated that the amino acid residues involved in the active site of neuraminidase in HPIV4 and their corresponding positions in the primary structure are as follows: R1: R175, R4: R417, R5: R507, Y6: Y535, E4: E402, E6: E556, and D1: D199 (Figure 5). To the best of our knowledge, these findings may also be novel.

Furthermore, selection pressure analysis was conducted to estimate host selection on the antigenic HN protein. The results indicated that while no positive selection sites were identified on the HPIV4 HN protein, relatively few negative selection sites were observed (fewer than nine sites). A previous report on the HPIV3 HN protein showed that some positive selection sites (five sites) were found in the strains [17]. Furthermore, many negative selection sites (18–160 sites) were estimated in the protein [17]. As a foreign antigen protein, it is subject to selection pressure due to the host’s immune defense responses [2]. Consequently, amino acid mutations in the antigen protein emerge as a means to evade this selection pressure [2,42]. Additionally, the presence of negative selection pressure suggests a role in maintaining the functional integrity of the protein [42,43]. Thus, it is possible that the HN protein of HPIV4 exhibits low antigenicity and is less susceptible to positive selection pressure from its human host [2]. Furthermore, the limited number of negatively selected sites suggests that the HN protein of HPIV4 may not require amino acid substitutions to maintain its functionality [2].

Additionally, to elucidate the relationship between the HN protein activity and con-formational epitopes on the HPIV4 HN protein, we constructed the 3D structure of the protein and mapped them onto it (Figure 6). As a result, active sites were located at the top and on multiple sides of the protein. It is suggested that the HN protein has two functions. Hemagglutinin (HA) facilitates the virus’s entry into host cells by binding to sialic acid receptors on the surface of the host cell, promoting viral uptake. Neuraminidase plays a role in the release of new viral particles from the host cell after infection [44]. By cleaving sialic acid, it allows newly formed viruses to detach from the cell surface and spread to other cells [45]. Thus, these two functions are critical for the virus’s ability to infect cells and propagate within the host [46]. Neutralizing antibodies target viral proteins, inhibiting receptor binding and protein activity, thereby preventing the spread of viral infection. These antibodies bind to specific epitopes in the head region of the HN protein, which inhibits hemagglutinin activity and blocks viral entry into host cells [47,48]. In this study, with the exception of strains belonging to HPIV4b/II, the conformational epitopes and active site of the HN protein generally coincided. It is well-known that antibodies binding near the active site of an enzyme typically inhibit its activity. Thus, the loss of function at the active sites, along with steric inhibition caused by antibodies, might prevent binding to substrates such as sialic acid, similar to the influenza virus [47,48]. On the other hand, a previous report on HPIV3 suggested that the active sites and conformational epitopes of the HN protein do not align well, which may contribute to reinfection [49]. Therefore, antibodies induced following HPIV4 infection may inhibit the HN activity of most HPIV4 strains, resulting in the prevention of HPIV4 reinfection. Thus, HPIV4 may be a pathogen less prone to reinfection compared to HPIV3. To the best of our knowledge, these findings may also be novel.

Finally, there are two possible biases in the results of this analysis. First, most of the virus strains used were isolated in a limited area located in the northern part of Japan, and thus may not be generalizable. Second, these virus strains were propagated in LLC-MK2 cells after isolation, and possible mutations in the viral genes may have occurred during culture, as has been reported in influenza viruses [50,51,52]. Further studies are warranted to address these concerns.

## Data Availability

The data presented in this study are available on request from the corresponding author.

## Supplementary Materials

The following supporting information can be downloaded at: https://www.mdpi.com/article/10.3390/microorganisms13020384/s1, Table S1: Primers used for PCR and sequencing; Table S2: Detailed data on the strains used in this study; Table S3: Catalytic and active site residues in influenza virus neuraminidase and *Paramyxoviridae* hemagglutinin-neuraminidase (HN). Reference [53] is cited in the supplementary materials.

## References

[1] Rima B., Balkema-Buschmann A., Dundon W.G., Duprex P., Easton A., Fouchier R., Kurath G., Lamb R., Lee B., Rota P. (2019). ICTV Virus Taxonomy Profile: Paramyxoviridae. J. Gen. Virol..

[2] Domingo E., Knipe D.M., Howley P.M. (2006). Virus Evolution. Fields Virology.

[3] Canchola J., Vargosko A.J., Kim H.W., Parrott R.H., Christmas E., Jeffries B., Chanock R.M. (1964). Antigenic Variation Among Newly Isolated Strains of Parainfluenza Type 4 Virus. Am. J. Hyg..

[4] Marx A., Gary H.E., Marston B.J., Erdman D.D., Breiman R.F., Török T.J., Plouffe J.F., File T.M., Anderson L.J. (1999). Parainfluenza virus infection among adults hospitalized for lower respiratory tract infection. Clin. Infect. Dis..

[5] Marx A., Török T.J., Holma R.C., Clarke M.J., Anderson L.J. (1997). Pediatric Hospitalizations for Croup (Laryngotracheobronchitis): Biennial Increases Associated with Human Parainfluenza Virus 1 Epidemics. J. Infect. Dis..

[6] Wang X., Li Y., Deloria-Knoll M., Madhi S.A., Cohen C., Arguelles V.L., Basnet S., Bassat Q., Brooks W.A., Echavarria M. (2021). Global burden of acute lower respiratory infection associated with human parainfluenza virus in children younger than 5 years for 2018: A systematic review and meta-analysis. Lancet Glob. Health.

[7] Russell E., Ison M.G. (2017). Parainfluenza Virus in the Hospitalized Adult. Clin. Infect. Dis..

[8] Bull J.J., Koelle K., Antia R. (2024). Waning immunity drives respiratory virus evolution and reinfection. bioRxiv.

[9] Branche A.R., Falsey A.R. (2016). Parainfluenza Virus Infection. Semin. Respir. Crit. Care Med..

[10] Marcink T.C., Englund J.A., Moscona A., Kslow R.A., Stanberry L.R., Le Duc J.W. (2014). Paramyxoviruses: Parainfluenza viruses. Viral Infections of Humans.

[11] Karron R.A., Collins P.L., Knipe D.M., Howley P.M. (2013). Parainfluenza viruses. Fields Virology.

[12] Branche A.R., Falsey A.R. (2016). Parainfluenza Virus Infection.

[13] Mizukoshi F., Kimura H., Sugimoto S., Kimura R., Nagasawa N., Hayashi Y., Hashimoto K., Hosoya M., Shirato K., Ryo A. (2024). Molecular Evolutionary Analyses of the Fusion Genes in Human Parainfluenza Virus Type 4. Microorganisms.

[14] Henrickson K.J. (2003). Parainfluenza Viruses. Clin. Microbiol. Rev..

[15] Coelingh K.L.v.W., Winter C.C., Jorgensen E.D., Murphy B.R. (1987). Antigenic and structural properties of the hemagglutinin-neuraminidase glycoprotein of human parainfluenza virus type 3: Sequence analysis of variants selected with monoclonal antibodies which inhibit infectivity, hemagglutination, and neuraminidase activities. J. Virol..

[16] Van Wyke Coelingh K.L., Winter C., Murphy B.R. (1985). Antigenic variation in the hemagglutinin-neuraminidase protein of human parainfluenza type 3 virus. Virology.

[17] Aso J., Kimura H., Ishii H., Saraya T., Kurai D., Nagasawa K., Matsushima Y., Ryo A., Takizawa H. (2020). Molecular evolution of the hemagglutinin-neuraminidase (HN) gene in human respirovirus 3. Virus Res..

[18] Caetano-Anollés G., Mokrousov I., Shitikov E. (2024). Chapter 1—Phylogenomic analysis and the origin and early evolution of viruses. Phylogenomics.

[19] Marques-Pereira C., Pires M., Moreira I.S., Shukla A.K. (2022). Chapter 8—Discovery of Virus-Host interactions using bioinformatic tools. Methods in Cell Biology.

[20] Mokrousov I., Shitikov E. (2024). Phylogenomics: Foundations, Methods, and Pathogen Analysis.

[21] Numazaki Y., Oshima T., Ohmi A., Tanaka A., Oizumi Y., Komatsu S., Takagi T., Karahashi M., Ishida N. (1987). A microplate method for isolation of viruses from infants and children with acute respiratory infections. Microbiol. Immunol..

[22] Aguilar J.C., Pérez-Breña M.P., García M.L., Cruz N., Erdman D.D., Echevarría J.E. (2000). Detection and identification of human parainfluenza viruses 1, 2, 3, and 4 in clinical samples of pediatric patients by multiplex reverse transcription-PCR. J. Clin. Microbiol..

[23] Abiko C., Mizuta K., Aoki Y., Ikeda T., Itagaki T., Noda M., Kimura H., Ahiko T. (2013). An outbreak of parainfluenza virus type 4 infections among children with acute respiratory infections during the 2011-2012 winter season in Yamagata, Japan. Jpn. J. Infect. Dis..

[24] Katoh K., Standley D.M. (2013). MAFFT Multiple Sequence Alignment Software Version 7: Improvements in Performance and Usability. Mol. Biol. Evol..

[25] Bouckaert R., Heled J., Kühnert D., Vaughan T., Wu C.H., Xie D., Suchard M.A., Rambaut A., Drummond A.J. (2014). BEAST 2: A software platform for Bayesian evolutionary analysis. PLoS Comput. Biol..

[26] Rambaut A., Drummond A.J., Xie D., Baele G., Suchard M.A. (2018). Posterior Summarization in Bayesian Phylogenetics Using Tracer 1.7. Syst. Biol..

[27] Kanda Y. (2013). Investigation of the freely available easy-to-use software ‘EZR’ for medical statistics. Bone Marrow Transplant..

[28] Kumar S., Stecher G., Tamura K. (2016). MEGA7: Molecular Evolutionary Genetics Analysis Version 7.0 for Bigger Datasets. Mol. Biol. Evol..

[29] Webb B., Sali A. (2021). Protein Structure Modeling with MODELLER. Methods Mol. Biol..

[30] Emsley P., Lohkamp B., Scott W.G., Cowtan K. (2010). Features and development of Coot. Acta Crystallogr. D Biol. Crystallogr..

[31] Guex N., Peitsch M.C. (1997). SWISS-MODEL and the Swiss-Pdb Viewer: An environment for comparative protein modeling. Electrophoresis.

[32] Zhou C., Chen Z., Zhang L., Yan D., Mao T., Tang K., Qiu T., Cao Z. (2019). SEPPA 3.0-enhanced spatial epitope prediction enabling glycoprotein antigens. Nucleic Acids Res..

[33] Liu W.K., Liu Q., Chen D.H., Liang H.X., Chen X.K., Huang W.B., Qin S., Yang Z.F., Zhou R. (2013). Epidemiology and clinical presentation of the four human parainfluenza virus types. BMC Infect. Dis..

[34] Takahashi T., Akagawa M., Kimura R., Sada M., Shirai T., Okayama K., Hayashi Y., Kondo M., Takeda M., Ryo A. (2023). Molecular evolutionary analyses of the fusion protein gene in human respirovirus 1. Virus Res..

[35] Feng Y., Zhu Z., Xu J., Sun L., Zhang H., Xu H., Zhang F., Wang W., Han G., Jiang J. (2023). Molecular Evolution of Human Parainfluenza Virus Type 2 Based on Hemagglutinin-Neuraminidase Gene. Microbiol. Spectr..

[36] Shao N., Liu B., Xiao Y., Wang X., Ren L., Dong J., Sun L., Zhu Y., Zhang T., Yang F. (2021). Genetic Characteristics of Human Parainfluenza Virus Types 1-4 from Patients with Clinical Respiratory Tract Infection in China. Front. Microbiol..

[37] Bose M.E., Shrivastava S., He J., Nelson M.I., Bera J., Fedorova N., Halpin R., Town C.D., Lorenzi H.A., Amedeo P. (2019). Sequencing and analysis of globally obtained human parainfluenza viruses 1 and 3 genomes. PLoS ONE.

[38] Smielewska A., Emmott E., Ranellou K., Popay A., Goodfellow I., Jalal H. (2018). UK circulating strains of human parainfluenza 3: An amplicon based next generation sequencing method and phylogenetic analysis. Wellcome Open Res..

[39] Mao N., Ji Y., Xie Z., Wang H., Wang H., An J., Zhang X., Zhang Y., Zhu Z., Cui A. (2012). Human parainfluenza virus-associated respiratory tract infection among children and genetic analysis of HPIV-3 strains in Beijing, China. PLoS ONE.

[40] Sharma S. (2017). Markov chain Monte Carlo methods for Bayesian data analysis in astronomy. Annu. Rev. Astron. Astrophys..

[41] Aso J., Kimura H., Ishii H., Saraya T., Kurai D., Matsushima Y., Nagasawa K., Ryo A., Takizawa H. (2020). Molecular evolution of the fusion protein (F) gene in human respirovirus 3. Front. Microbiol..

[42] Ngandu N.K., Scheffler K., Moore P., Woodman Z., Martin D., Seoighe C. (2008). Extensive purifying selection acting on synonymous sites in HIV-1 Group M sequences. Virol. J..

[43] Farzadfar S., Pourrahim R. (2019). Positive selection and recombination shaped the large genetic differentiation of Beet black scorch virus population. PLoS ONE.

[44] Su B., Wurtzer S., Rameix-Welti M.-A., Dwyer D., van Der Werf S., Naffakh N., Clavel F., Labrosse B. (2009). Enhancement of the influenza A hemagglutinin (HA)-mediated cell-cell fusion and virus entry by the viral neuraminidase (NA). PLoS ONE.

[45] Burzyńska P., Sobala Ł.F., Mikołajczyk K., Jodłowska M., Jaśkiewicz E. (2021). Sialic acids as receptors for pathogens. Biomolecules.

[46] Iorio R.M., Syddall R.J., Sheehan J.P., Bratt M.A., Glickman R.L., Riel A.M. (1991). Neutralization map of the hemagglutinin-neuraminidase glycoprotein of Newcastle disease virus: Domains recognized by monoclonal antibodies that prevent receptor recognition. J. Virol..

[47] Brandenburg B., Koudstaal W., Goudsmit J., Klaren V., Tang C., Bujny M.V., Korse H.J., Kwaks T., Otterstrom J.J., Juraszek J. (2013). Mechanisms of hemagglutinin targeted influenza virus neutralization. PLoS ONE.

[48] Zhang Y., Xu C., Zhang H., Liu G.D., Xue C., Cao Y. (2019). Targeting Hemagglutinin: Approaches for Broad Protection against the Influenza A Virus. Viruses.

[49] Suryadevara N., Otrelo-Cardoso A.R., Kose N., Hu Y.-X., Binshtein E., Wolters R.M., Greninger A.L., Handal L.S., Carnahan R.H., Moscona A. (2024). Functional and structural basis of human parainfluenza virus type 3 neutralization with human monoclonal antibodies. Nat. Microbiol..

[50] Li D., Saito R., Suzuki Y., Sato I., Zaraket H., Dapat C., Caperig-Dapat I.M., Suzuki H. (2009). In vivo and in vitro alterations in influenza A/H3N2 virus M2 and hemagglutinin genes: Effect of passage in MDCK-SIAT1 cells and conventional MDCK cells. J. Clin. Microbiol..

[51] Lee H.K., Tang J.W.-T., Kong D.H.-L., Loh T.P., Chiang D.K.-L., Lam T.T.-Y., Koay E.S.-C. (2013). Comparison of mutation patterns in full-genome A/H3N2 influenza sequences obtained directly from clinical samples and the same samples after a single MDCK passage. PLoS ONE.

[52] Chambers B.S., Li Y., Hodinka R.L., Hensley S.E. (2014). Recent H3N2 influenza virus clinical isolates rapidly acquire hemagglutinin or neuraminidase mutations when propagated for antigenic analyses. J. Virol..

[53] Lawrence M.C., Borg N.A., Streltsov V.A., Pilling P.A., Epa V.C., Varghese J.N., McKimm-Breschkin J.L., Colman P.M. (2004). Structure of the Haemagglutinin-neuraminidase from Human Parainfluenza Virus Type III. J. Mol. Biol..

