# Supplementary material for: Phylogenomic Analyses of the Hemagglutinin-Neuraminidase (HN) Gene in Human Parainfluenza Virus Type 4 Isolates in Japan
Source: Microorganisms. 2025 Feb 10;13(2):384. doi: 10.3390/microorganisms13020384 (PMC11857914; doi:10.3390/microorganisms13020384)
Supplement: Supplementary file 1 [file microorganisms-13-00384-s001.zip › Table S1 Primers used for PCR and sequencing..pdf]

**Supplementary Table S1** Primers used for PCR and sequencing. Some primers were modified from Abiko *et.al*, Jpn J Infect Dis. 2013.

| Primer name | Sequence | Number of bases |
|---|---|---|
| **F1** | CAACAATCCAGARRGACGTCACA | 822bp |
| **R3-721** | ATTTAAATGAYRGGARGAATCG | |
| **F4** | CCRTCATTCTCCYTARGTCAAA | 764bp |
| **R5** | CCHTATCCRAGYCTYTATAGAATYA | |
| **F5** | TGCAGAGGGTCGMCTATATAATATTG | 540bp |
| **R6** | AKTRAYAGYCTGRTCTATTRAG | |

Abiko C, Mizuta K, Aoki Y, Ikeda T, Itagaki T, Noda M, Kimura H, Ahiko T. An outbreak of parainfluenza virus type 4 infections among children with acute respiratory infections during the 2011-2012 winter season in Yamagata, Japan. Jpn J Infect Dis. 2013;66(1):76-8.

doi: 10.7883/yoken.66.76.
https://www.jstage.jst.go.jp/article/yoken/66/1/66_76/_article
