# Supplementary material for: Phylogenomic Analyses of the Hemagglutinin-Neuraminidase (HN) Gene in Human Parainfluenza Virus Type 4 Isolates in Japan
Source: Microorganisms. 2025 Feb 10;13(2):384. doi: 10.3390/microorganisms13020384 (PMC11857914; doi:10.3390/microorganisms13020384)
Supplement: Supplementary file 1 [file microorganisms-13-00384-s001.zip › Table S2 Detailed data on the strains used in this study_20250206.pdf]

Table S2 Detailed data on the strains used in this study.

Accession Number	Year	Strain
MH892407	2013	
KY460515	2010	
EU627591	2004	
MN306032	2019	
LC862243	2002	02_3095_Fukuoka
LC862244	2002	02_3269_Fukuoka
LC862171	2002	02_4394
LC862172	2003	03_3688
LC862173	2003	03_3791
LC862174	2003	03_3852
LC862175	2003	03_3898
LC862176	2003	03_3935
LC862177	2003	03_4105
LC862178	2003	03_4220
LC862179	2003	03_4617
LC862180	2003	03_N1046
LC862181	2003	03_N1056
LC862182	2004	04_1960_Miyagi
LC862183	2004	04_2608
LC862184	2004	04_2830
LC862185	2004	04_2853
LC862186	2004	04_3034
LC862187	2004	04_3057
LC862188	2004	04_3132
LC862189	2004	04_3202
LC862190	2004	04_3276
LC862191	2004	04_3340
LC862192	2004	04_3354
LC862193	2004	04_3365
LC862194	2004	04_3430
LC862195	2004	04_3439
LC862245	2004	04_K313_Yamagata
LC862196	2004	04_K373

LC862246	2004	04_K435_Yamagata
LC862247	2004	04_K448_Yamagata
LC862197	2004	04_N1027
LC862198	2004	04_N116
LC862199	2004	04_N856
LC862200	2004	04_N967
LC862248	2006	06_K347_Yamagata
LC862249	2006	06_K370_Yamagata
LC862250	2006	06_K375_Yamagata
LC862201	2006	06_N781
LC862202	2007	07_1508
LC862203	2007	07_1969
LC862204	2007	07_2012
LC862205	2007	07_2096
LC862251	2007	07_K10_Yamagata
LC862206	2007	07_N806
LC862207	2007	07_N816
LC862208	2007	07_N872
LC862209	2007	07_N873
LC862210	2007	07_N893
LC862211	2008	08_1475
LC862212	2008	08_1797
LC862213	2008	08_1840
LC862214	2008	08_1849
LC862215	2008	08_1906
LC862216	2008	08_2048
LC862217	2008	08_N507
LC862218	2009	09_N795
LC862219	2011	11_1373
LC862220	2011	11_1388
LC862252	2011	11_1393_Tokyo
LC862221	2011	11_1419
LC862222	2011	11_1467
LC862223	2011	11_1469
LC862253	2011	11_1490_Tokyo
LC862224	2011	11_1496

LC862254	2011	11_1535_Fukuoka
LC862255	2011	11_1540_Fukuoka
LC862268	2011	11_2132_Yamagata
LC862269	2011	11_2238_Yamagata
LC862270	2011	11_2265_Yamagata
LC862271	2011	11_2269_Yamagata
LC862272	2011	11_2376_Yamagata
LC862273	2011	11_2378_Yamagata
LC862274	2011	11_2543_Yamagata
LC862275	2011	11_2555_Yamagata
LC862276	2011	11_2620_Yamagata
LC862277	2011	11_2628_Yamagata
LC862278	2011	11_2629_Yamagata
LC862279	2011	11_2679_Yamagata
LC862256	2011	11_K268_Yamagata
LC862257	2011	11_K325_Yamagata
LC862258	2011	11_K360_Yamagata
LC862225	2011	11_N610
LC862226	2011	11_N663
LC862227	2011	11_N732
LC862228	2011	11_N734
LC862229	2012	12_1289
LC862263	2012	12_149_Yamagata
LC862230	2013	13_1013
LC862231	2013	13_1119
LC862232	2013	13_1182
LC862233	2013	13_1230
LC862234	2013	13_1259
LC862235	2013	13_1287
LC862236	2013	13_1413
LC862237	2013	13_1417
LC862259	2013	13_K342_Yamagata
LC862260	2013	13_K346_Yamagata
LC862261	2013	13_K348_Yamagata
LC862262	2013	13_K368_Yamagata
LC862238	2014	14_1255

LC862239	2014	14_928
LC862264	2014	14_K338_Yamagata
LC862240	2015	15_1326
LC862241	2015	15_665
LC862242	2015	15_818
LC862265	2015	15_K135_Yamagata
LC862266	2015	15_K224_Yamagata
LC862267	2015	15_K353_Yamagata
