# Supplementary material for: Phylogenomic Analyses of the Hemagglutinin-Neuraminidase (HN) Gene in Human Parainfluenza Virus Type 4 Isolates in Japan
Source: Microorganisms. 2025 Feb 10;13(2):384. doi: 10.3390/microorganisms13020384 (PMC11857914; doi:10.3390/microorganisms13020384)
Supplement: Supplementary file 1 [file microorganisms-13-00384-s001.zip › Table S3 Catalytic and active site residues in influenza virus neuraminidase and paramyxoviridae HN.pdf]

Table S3. Catalytic and active site residues in influenza virus neuraminidase and *Paramyxoviridae* hemagglutinin-neuraminidase (HN).

Residue	Influenza virus neuraminidase	PIV3 HN	PIV4 HN	NDV HN
R1	118	192	175	174
R4	292	424	417	416
R5	371	502	507	498
Y6	406	530	535	526
E4	277	409	402	401
E6	425	549	556	547
D1	151	216	199	198

Reference

1. Lawrence, M. C., Borg, N. A., Streltsov, V. A., Pilling, P. A., Epa, V. C., Varghese, J. N., McKimm-Breschkin, J. L., & Colman, P. M. (2004). Structure of the Haemagglutinin-neuraminidase from Human Parainfluenza Virus Type III. Journal of Molecular Biology, 335(5), 1343-1357. https://doi.org/10.1016/j.jmb.2003.11.032
